# Construction and Characterization of a Humanized Anti-Epstein-Barr Virus gp350 Antibody with Neutralizing Activity in Cell Culture

**DOI:** 10.3390/cancers10040112

**Published:** 2018-04-09

**Authors:** Jerome E. Tanner, Jing Hu, Caroline Alfieri

**Affiliations:** 1 Laboratory of Viral Pathogenesis, Research Centre, CHU Sainte-Justine, Montréal, QC H3T 1C5, Canada; jetanner@videotron.ca (J.E.T.); jing.hu@recherche-ste-justine.qc.ca (J.H.); 2 Department of Microbiology, Infectiology and Immunology, University of Montreal, 3175 Côte Ste-Catherine Road, Montreal, QC H3T 1C5, Canada

**Keywords:** Epstein-Barr virus, humanized antibody, glycoprotein 350, glycoprotein 220, homology modeling, complementarity determining region

## Abstract

Acute Epstein-Barr virus (EBV) infection in immunosuppressed transplant patients can give rise to a malignant B-cell proliferation known as post-transplant lymphoproliferative disease (PTLD). The EBV major virion surface glycoprotein (gp)350 is a principal target of naturally occurring neutralizing antibodies and is viewed as the best target to prevent acute infection and PTLD in at-risk transplant recipients. We have constructed a humanized (hu) version of the murine anti-gp350 neutralizing monoclonal antibody 72a1. The hu72a1 IgG1 antibody displayed no significant anti-mouse activity, recognized both gp350 and its splice variant gp220 as well as a gp350 peptide that was shown to constitute the principal EBV gp350 neutralizing epitope when tested in immunoassays. Hu72a1 antibody blocked in vitro EBV infection of B cells at a level which equaled that of a mouse-human chimeric 72a1 antibody construct. This work provides a further structural and immunological understanding of the 72a1 antibody interaction with EBV gp350, and constitutes a launch point for future anti-EBV therapeutic antibodies designed to block EBV infection and prevent PTLD while eliminating the deleterious antigenic murine features of the original 72a1 antibody.

## 1. Introduction

The Epstein-Barr virus (EBV), a member of the *Gammaherpesvirinae* subfamily, has the unique biological property of immortalizing human B lymphocytes for continuous proliferation in cell culture [1]. A healthy immune system maintains EBV in a latent persistent state by blocking virus replication and suppressing the growth of infected B cells. However, when the immune system is suppressed, as in transplant patients treated with drugs to prevent graft rejection [2], B cells harbouring EBV may become growth-unrestrained and give rise to the malignant B cell lymphoproliferation known as post-transplant lymphoproliferative disease (PTLD) [3,4]. A well-recognized high-risk factor for PTLD is the occurrence of primary EBV infection while the organ transplant recipient is undergoing intense immunosuppressive treatment [5]. Children are particularly prone to acquiring primary EBV infection and subsequent PTLD (up to 30% incidence) due to their higher likelihood of being EBV seronegative (i.e., EBV naïve) at the time of organ transplant compared to adults [6,7,8]. This absence of prior EBV exposure is the main reason for the increased prevalence of PTLD in the pediatric transplant setting [9,10]. It follows that with the increasing use of the transplantation option in children, PTLD and other EBV-associated B-cell lymphoma are now the most common non-Hodgkin’s lymphoma seen in the pediatric transplant population [11].

Attempts to manage EBV disease in transplant patients usually involve strategies to treat the disease well after the virus is entrenched [2,4]. Common treatment strategies include decreasing levels of immune suppressive medications, administering chemotherapy or treating with Rituximab, an anti-CD20 monoclonal that purges mature B cells from the patient’s blood and tissues [12,13]. These reactionary approaches begin only after elevated levels of EBV-infected B cells are detected in the blood, and may actually promote EBV replication [14,15,16,17], infection morbidity [18] or lead to more aggressive forms of PTLD [12,19].

A proactive strategy that does not rely on the host’s compromised immune system to prevent infection would constitute an effective means to prevent PTLD. The EBV major virion surface glycoprotein gp350 is the principal target of naturally occurring neutralizing antibodies, and is considered the best target to prevent acute EBV infection and PTLD in at-risk solid organ and hematopoietic stem cell recipients [20,21]. Antibodies directed against gp350 were shown to prevent B-cell immortalization in culture [22,23,24] and B-cell lymphoma in a small primate model [25,26,27,28,29]. Monoclonal antibody epitope mapping of gp350 points to a dominant and highly conserved virus neutralizing epitope [30]. Current attempts to enhance the patients’ natural immunity through gp350 vaccination [31] or through passive boosting of humoral immunity with intravenous immune globulin (IVIG) have proven ineffective [32]. Perhaps a more effective approach would be to temporally increase the serum level of anti-EBV neutralizing antibody early post-transplant when the patient is most at risk of acute EBV infection and PTLD.

We have designed a humanized (hu) version of the well-characterized EBV-neutralizing murine monoclonal antibody 72a1 [24], and show that this hu72a1 monoclonal antibody can recognize gp350 and gp220, block in vitro virus infection of B cells, while also demonstrating no significant anti-mouse immunoreactivity.

## 2. Results

### 2.1. Humanization and Anti-Mouse Immunogenicity Testing of Anti-EBV gp350 Monoclonal Antibody 72a1

Prior to undertaking the humanization of mouse (m)72a1, we re-examined the reported differences in anti-gp350 reactivity observed with the use of antibody constructs based on two separate sets of m72a1 variable region (VR) cDNAs [33,34]. An examination of archived chimeric antibody samples generated in our laboratory prior to our earlier publication showed that the strongest anti-gp350 immunoreactivity was found in a chimeric antibody based on GenBank cDNA sequences KT211017.1 and KT211018.1 (Figure S1) [34].

**Figure 1:**
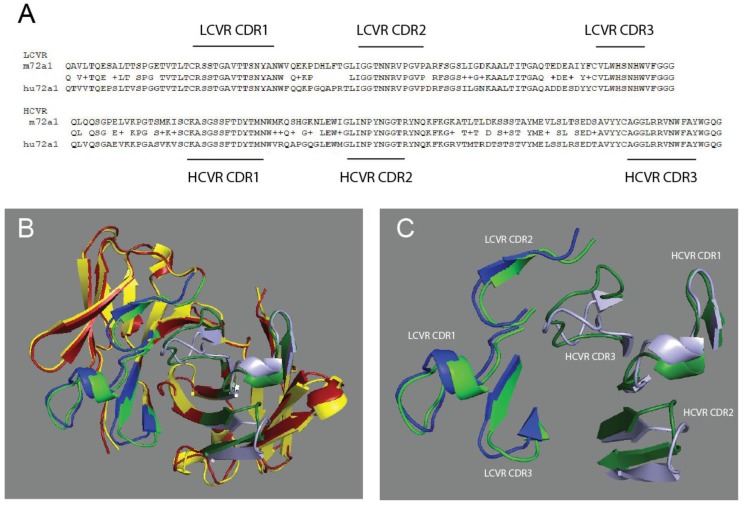
Comparison of m72A1 and hu72a1 heavy chain (HC) and light chain (LC) variable region (VR) sequences. (**A**) Amino acid sequence alignment and complementarity-determining region (CDR) assignment for m72a1 and hu72a1 VR; (**B**) Spatial depiction of PyMOL alignments for lowest energy Rosetta antibody VR models; m72a1 framework (yellow), m72a1 CDRs (green), hu72a1 framework (red) and hu72a1 CDRs (blue); (**C**) Spatial depiction of m72a1 HC CDRs (dark green) and LC CDR (light green). Spatial depiction of hu72a1 LC CDR (dark blue) and HC CDR (pale blue).

m72a1 antibody humanization began with a BLAST analysis of m72a1 VR sequence similarity to the human heavy chain (HC), light chains (LC) and antibody germline sequences listed in the NCBI database [35]. Criteria for the selection and alteration of a given m72a1 VR framework sequence were based on preserving the original m72a1 complementarity determining regions (CDR) and their neighbouring amino acid sequences [36]. The final humanized 72a1 antibody design showed no residual murine-specific Ig sequences and 100% identity to human antibody framework sequences by BLAST analysis (Figure 1A). Rosetta antibody modeling was used to ascertain the predicted hu72a1 VR (including CDRs) structure and compare it to a model structure generated by the m71a1 VR sequences (Figure 1B,C).

An earlier exploratory clinical study using m72a1 to prevent EBV infection in at-risk transplant patients resulted in all four patients developing anti-mouse 72a1 antibodies, likely triggered by the patient’s immune system response to m72a1 as a foreign antigen [37,38]. We examined the levels of anti-mouse and anti-human activity retained in our hu72a1 by ELISA. m72a1 and hu72a1 were tested for their respective reactivity to species affinity-purified goat anti-mouse (GAM) IgG and species affinity-purified goat anti-human (GAH) IgG.

**Figure 2:**
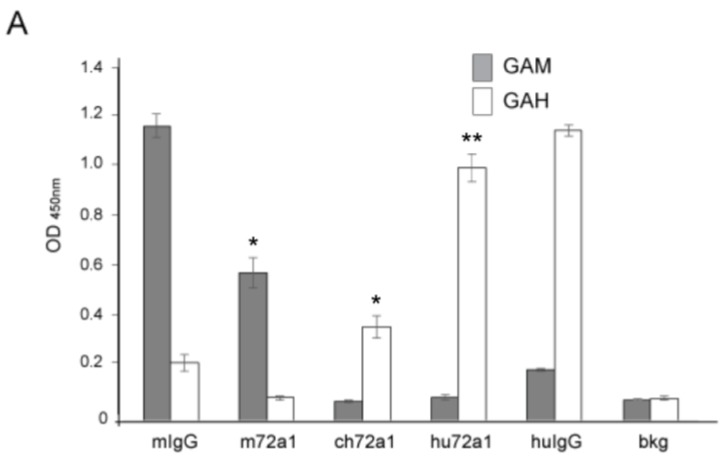

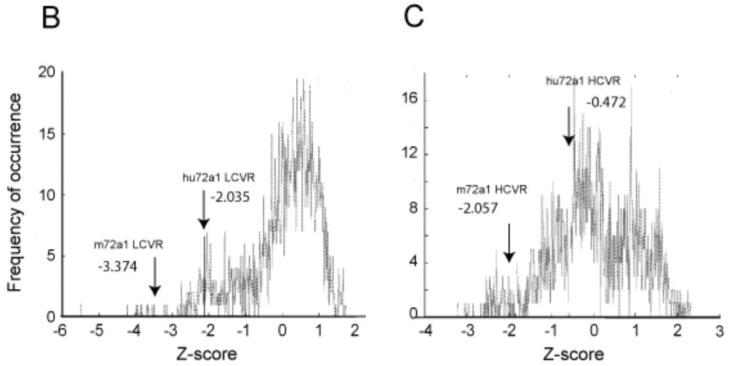
Hu72a1 anti-mouse antibody reactivity and relative Ig “humanness”. (**A**) Average OD_450nm_ values ± SD for goat anti-mouse IgG (GAM, closed box) or goat anti-human IgG (GAH, open box) reactivity for mouse IgG (mIg), murine 72a1 (m72a1), chimeric 72a1 (ch72a1), human 72a1 (hu72a1) or ELISA coating buffer activity (background, bkg). Barr plot averages and SD were derived from triplicate samples and generated from two separate experiments; (**B**) Z-score plot of m72A1 and hu72a1 LC VR; (**C**) Z-score plot of m72a1 and hu72a1 HC VR. Z-score value locations within the human Ig repertoire are indicated by an arrow. T-test probability (*p*) values ≤ 0.01 * and ≤ 0.001 ** are shown.

As shown in Figure 2A, m72a1 reacted strongly to GAM (7-fold. *p* = 0.005) as compared to background (bkg) OD levels but did not react to GAH (0.4-fold over bkg *p* = 0.7) (Figure 2A). Conversely, chimeric (ch)72a1 and hu72a1 did not react significantly to GAM (0 and 0.1-fold over bkg, *p* = 0.3 and *p* = 0.2 respectively, Figure 2A), but did react 4-fold (ch72a1) and 11-fold (hu72a1) to GAH IgG compared to bkg (*p* = 0.006 and *p* = 0.001, respectively, Figure 2A). These results indicate that humanization of m72a1 significantly reduced anti-mouse reactivity and increased anti-human reactivity.

Further comparison of the VR sequences for m72a1 and hu72a1 to human VR sequences listed in the Kabat and KabatMan databases using the SHAB web server program [39] showed that our chosen amino acid alterations in the final 72a1 antibody design gave the original m72a1 VR sequences a more human appearance. Based on the percentage of human Ig sequence identity, the SHAB program generates a Z-score reflecting relative relatedness of a particular antibody sequence to the human Ig repertoire. A Z-score of zero represents an average similarity to the entire human VR repertoire. A positive Z-score indicates that a given VR sequence is more common in the human Ig repertoire. Conversely, a negative Z-score indicates that the VR sequence is less common in the human Ig repertoire [39]. Comparison of m72a1 HC and LC VR Z-scores to those seen with hu72a1 showed that humanization positively increased LC VR “humanness” by 1.3 ≤ σ (−3.374 m72a1 lambda LC VR versus −2.035 hu72a1 lambda LC VR) (Figure 2B). Similarly, humanization of m72a1 HC VR positively increased HC VR “humanness” by 1.6σ (−2.057, m72a1 HC VR versus −0.472 hu72a1 HC VR) (Figure 2C). The Z-score value generated by the SHAB program for hu72a1 agrees well with other therapeutic antibodies which underwent a similar humanization process and whose Z-scores ranged from −1.8 to 0.46 [39]. These results indicate that humanization of m72a1 resulted in a significant reduction in mouse immunoreactivity and an increase in 72a1 “humanness”.

### 2.2. Recognition of gp350 and gp220 by Humanized 72a1

**Figure 3:**
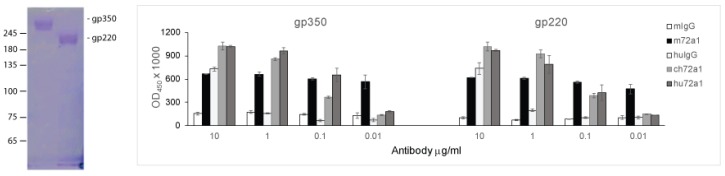
Hu72a1 recognizes gp350 and gp220 in ELISA. (**Left**) A 6% polyacrylamide SDS gel of purified gp350 and gp220 used in the ELISA; (**Right**) OD_450nm_ values ± SE were derived from triplicate samples for each antibody concentration, and the plotted average was calculated from three separate experiments.

In order to verify that hu72a1 antibody maintained its sensitivity-detection of gp350 when present in low antibody concentrations, log_10_ dilutions ranging from 10 to 0.01 μg/mL for mouse, chimeric and human 72a1 antibody were tested by ELISA using purified gp350 or gp220 as target analytes. As shown in Figure 3, ch72a1 and hu72a1 antibodies were capable of detecting gp350 and gp220 at an antibody concentration ≥0.1 μg/mL. Ch72a1 exhibited a 6-fold (*p* = 0.0009) and 4-fold (*p* = 0.001) anti-gp350 and anti-gp220 immunoreactivity versus huIgG, respectively (Figure 3). Hu72a1 exhibited a 10-fold (*p* = 0.006) and 4-fold (*p* = 0.04) anti-gp350 and anti-gp220 immunoreactivity versus huIgG, respectively (Figure 3). ELISA data also indicated that m72a1 was able to detect gp350 and gp220 at an antibody concentration ≥0.01 μg/mL. m72a1 exhibited a 4-fold (*p* = 0.02) and 4-fold (*p* = 0.01) anti-gp350 and anti-gp220 immunoreactivity versus mIgG, respectively (Figure 3). Since ch72a1 and m72a1 share identical VR sequences, the observed 10-fold increase in sensitivity to detect gp350 and gp220 by m72a1 antibody may be explained by the notion that placement of mVR sequences adjacent to a huIg constant region may yield changes in antibody folding that distally influences m72a1 CDR shape and affinity for gp350 [40].

**Figure 4:**
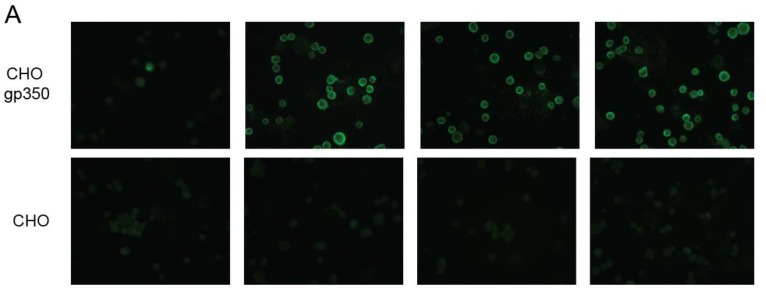

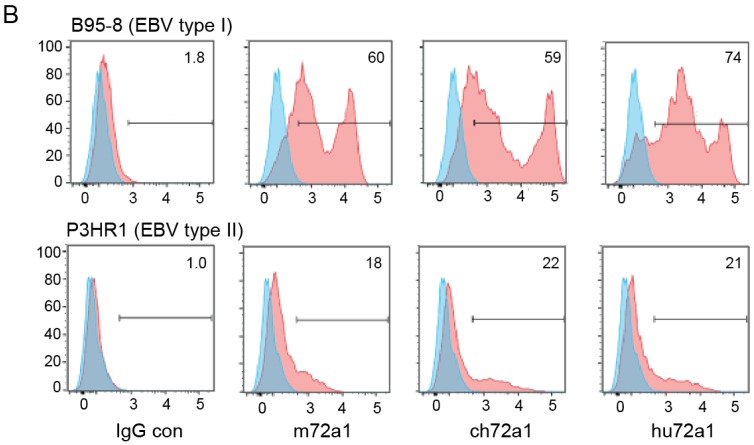
Hu72a1 antibody recognizes native gp350. (**A**) FACS analysis of type 1 gp350 (B95-8) and type 2 gp220 (P3HR1) undergoing lytic infection and stained with isotype-matched control (IgG con), m72a1, ch72a1 or hu72a1; (**B**) m72a1, ch72a1 and hu72a1 antibody immunofluorescence of gp350 and gp220 expressed on the surface of CHO cell clone 5.20 (CHO gp350) or parental CHO-K1 cell line.

To further assess the hu72a1 antibody capacity to recognize gp350 and gp220 native structures, we performed immunofluorescence assays using live B95-8 and P3HR-1 cells (FACS) and fixed CHO cells (microscopy). Live cell immunofluorescence of B95-8 and P3HR-1 cells undergoing lytic infection enabled us to measure the ability of hu72a1 to detect type I or type II virus forms of gp350, respectively [41]. CHO cells engineered to express only gp350 and gp220 on their cell surface allowed us to examine gp350 binding without the interference of other virion glycoproteins. As shown in Figure 4A, m72a1, ch72a1 and hu72a1 recognized gp350 when expressed on the CHO cell surface. Further analysis of type 1 and type 2 virus gp350 expression by FACS showed that mouse, chimeric and human forms of 72a1 comparably recognized the percentage of cells expressing surface type 1 and type 2 gp350 (gp350 type 1: 60% for m- and ch72a1 versus 74% for hu72a1; gp350 type 2: 18–22% for m-, ch- and hu72a1) (Figure 4B). We noted that hu72a1 appeared to detect a higher portion of cells expressing moderate amounts of gp350 type 1 and a smaller portion of cells expressing high amounts of gp350 type 1 (Figure 4B, B95-8) as compared to ch72a1 or m72a1 (Figure 4B, B95-8). This suggests that hu72a1 might recognize a subspecies of type 1 gp350 or that it recognizes gp350 type 1 differently compared to ch72a1 or m72a1.

### 2.3. Recognition of gp350 Mimetic Peptides by hu72a1

**Figure 5:**
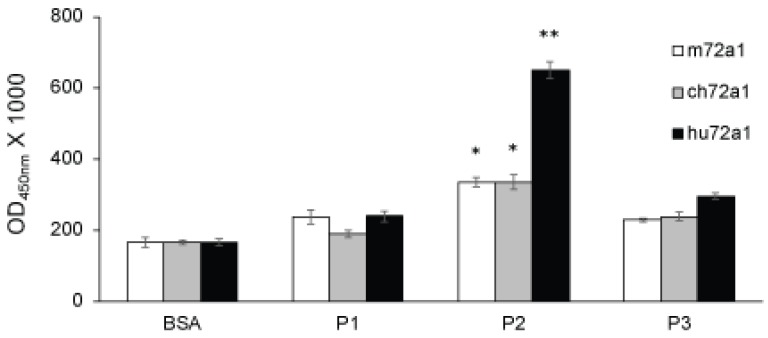
72a1 specifically binds gp350 mimetic peptide 2 [33]. OD_450nm_ values ± SE were derived from triplicate samples and from three separate experiments. *T*-test probability (*p*) values ≤ 0.01 * and ≤ 0.001 ** are shown.

Previous mapping of the 72a1 antibody virus neutralizing epitope localized it to the amino-terminal domain of gp350 [42,43,44]. Fine-mapping of the virus neutralizing epitope further identified gp350 sequences _22_DDRTLQIL_15_ and _148_NPVYLIPETVPYIKWDN_164_ and was referred to as gp350 mimetic peptide 2 (P2) [33]. Examination of hu72a1’s ability to recognize P2 by ELISA has revealed that hu72a1 bound 2.5-fold (P1 vs. P2, *t*-test *p* = 0.0005) more to P2 as compared to a gp350 peptide sequence shown not to be involved in m72a1 binding (P1) [44] or to a peptide located within the planar domain of gp350 involved in receptor binding (P3) (Figure 5) [33]. Although to a lesser extent, P2 was also uniquely recognized by m72a1 and ch72a1. m72a1 and ch72a1 bound to P2 1.5-fold and 1.6-fold more than P1 or P3 (P1 vs. P2 *p* = 0.002; P1 vs. P3 *p* = 0.006, respectively) (Figure 5). These results re-affirm the notion that P2 sequences _22_DDRTLQIL_15_ and _148_NPVYLIPETVPYIKWDN_164_ are recognized by 72a1 antibody and constitute a significant component of the gp350 neutralizing epitope.

### 2.4. Hu72a1 Blocks In Vitro EBV Infection

Experimental evidence indicates that m72a1 neutralizes EBV by blocking its attachment to the EBV B-cell receptor CD21 [43,45]. To determine whether hu72a1 could block EBV attachment to its B-cell receptor, P3HR-1 virus was incubated with equal amounts of hu72a1 or ch72a1 antibody prior to virus infection of Raji cells. As shown in Table 1, at a concentration of 1 μg/mL chimeric and human 72a1 antibodies inhibited virus infection 73.9% ± 14.6 SD and 79.0% ± 9.5 SD, respectively. The degree of virus neutralization using 1 μg/mL is in agreement with earlier findings for m72a1 [43].

### 2.5. Fine Mapping of hu72a1 Interaction with gp350

**Figure 6:**
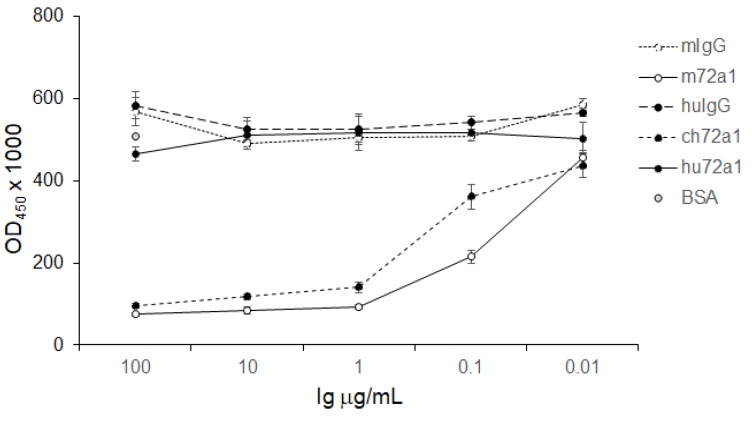
72a1 competition ELISA. Log_10_ dilutions of purified m72a1, ch72a1 and hu72a1 antibody were incubated with purified gp350 for 1 h prior to the addition of a 1:3000 dilution of a 1 mg/mL stock solution of biotin-labeled m72a1. Plots of average OD_450nm_ values ± SD were determined based on readings from triplicate samples.

We reasoned that if hu72a1 binds in both strength and epitope identity to gp350 as does m72a1, one could expect that hu72a1 would compete effectively with a tagged version of m72a1 for gp350 recognition and binding [33]. Data from a competition ELISA assay, whereby log_10_ dilutions of m72a1, ch72a1 or hu72a1 were incubated with gp350 followed by addition of biotin-labeled m72a1, revealed that m72a1 and ch72a1 successfully blocked tagged m72a1 from binding to gp350 at antibody levels ≥1 μg/mL. Surprisingly, hu72a1 was unable to block binding by the tagged m72a1 even when used at 100 μg/mL (Figure 6). These results, together with the binding recognition of Peptide 2 (Figure 5) and comparable virus neutralizing potencies of human and chimeric 72a1 (Table 1), suggest that hu72a1 and m72a1 virus neutralization is not contingent on a complete or identical recognition of gp350 as depicted by the m72a1 VR structure.

**Figure 7:**
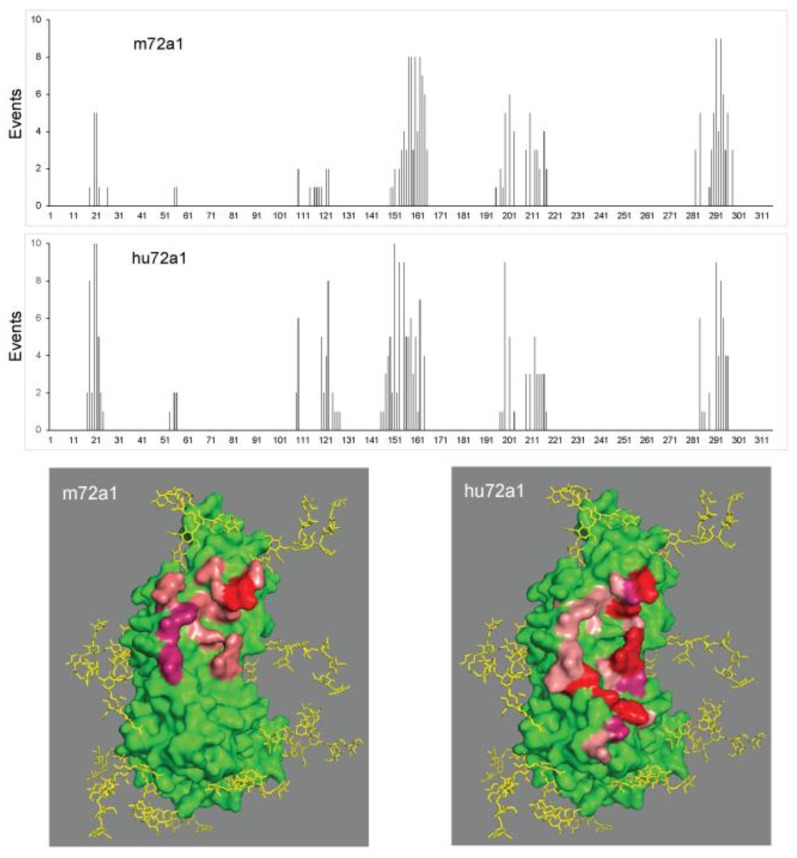
Frequency map of gp350 amino acid polar contacts with m72a1 VR or hu72a1 VR. (Upper two panels) Plots of polar contact events for gp350 amino acids 1 to 315 were based on the 10 best (lowest) Rosetta Docking 2 energy interface models between gp350 crystal structure and mouse or human 72a1 VR models. (Lower two panels) Structural models of gp350 amino acid polar contact events with mouse or human 72a1. gp350 (green), salmon (5–7:10 contact events), purple (8:10 contact events) and red (9–10:10 contact events).

In order to posit whether m72a1 and hu72a1 bound differently to gp350 we performed Rosetta Docking 2 analysis using the published gp350 crystal structure [42] and our Rosetta antibody structural models for m72a1 and hu72a1. From Rosetta Docking of the 10 best (lowest energy) interface models, an event map of individual gp350 amino acid polar contacts (≤6 Angstroms, Å) with hu72a1 or m72a1 antibodies was generated. As summarized in Figure 7 (top two panels), we noted an overall similarity in event numbers and location for gp350 amino acids which showed polar contact with human and mouse 72a1. Interestingly, three of the four principal regions of gp350 amino acid polar contacts, centered at gp350 amino acids 20, 154 and 293, are also found in the gp350 neutralization region encompassed by mimetic peptides P2 and P3 [33]. The region of gp350 centered at amino acid 212 and showing polar contacts with m72a1 or hu72a1 was previously shown by mutation analysis to affect m72a1 recognition [42]. We also observed that hu72a1 antibody models predicted a unique set of amino acid contacts (underlined) located in gp350 peptide sequence 101-ELALTMRSKKLPINITTGEEQQVSLESVDV-130 that were less apparent (Lys 109, 6:10 hu72a1 models vs 2:10 m72a1) or not seen (Glu 120, 8:10 hu72a1 models vs 0:10 m72a1 models) (Figure 7). A graphic model of gp350 polar contacts wherein 6–10:10 events occurred in our m72a1 and hu72a1 further illustrates that m72a1 contact interaction is at an upper region of gp350’s amino acid planar domain, whereas hu72a1 contact interaction is more broadly dispersed.

### 2.6. Spatial Comparison of hu72a1 and m72a1 CDRs

From the predicted differences in the amino acid contacts between gp350 and m72a1 or hu72a1 (Figure 7), we assessed whether the predicted spatial distances between or within the human and mouse 72a1 CDRs had changed upon humanization of m72a1. We examined the predicted distances between each of the six CDRs, the distance between CDR-loop-anchor sequences and the angular trajectory of individual CDR loops for m72a1 or hu72a1 [46]. As shown in Figure 1 and detailed in Table 2, insertion of m72a1 CDRs into a human VR framework decreased the apparent distance between hu72a1 HC CDR2 and LC CDR3 by three H-bond distances (~6 Å) (Table 2). The decrease in distance could change how hu72a1 CDR2 or CDR3 interface with gp350 as compared to m72a1.

We noted that m72a1 HC CDR1 → HC CDR2 H-bond units decreased by two H-bond distances (~4 Å) in hu72a1. This may change how HC CDR2 or LC CDR3 interact with gp350. Such distance alteration may cause a H-bond to change from one that is mostly covalent to one that is mostly electrostatic.

An examination of the CDR-loop-anchor distances and CDR-loop angular trajectory showed that spatial angles for LC CDR2 and HC CDR1 might have changed by 10 and 7.4 degrees, respectively (Table 3). Lastly, the HC CDR3 loop which formed an open bulge-loop structure in m72a1 is now distorted such that the tandem arginine in the HC CDR3 sequence AGGLRRVNWFAY may have shifted from the loop apex to the rightward side of the loop (Figure 1B). These findings suggest that, independent of CDR peptide sequence, the VR framework environment could significantly contribute to 72a1 interaction with gp350.

## 3. Discussion

The goal in cancer therapy is to deploy drugs capable of exclusively destroying disseminated tumour cells while sparing normal tissue. With this aim, major efforts have been directed at harnessing the exquisite specificity of monoclonal antibodies [47]. Widening the application of antibody therapy to cancer treatment has been slowed because of two important issues: first, a requirement for the antibody to interact with human Fc receptors or complement and, second, the fact that mouse antibodies are immunogenic in the human system thereby eliciting human anti-mouse antibody responses (HAMA). This latter issue is known to induce anaphylactic shock and to weaken antibody potency with repeated administrations [38,48,49].

The impact of these two issues is seen in a pilot study to treat at-risk EBV-seronegative children with m72a1. Although providing beneficial short-term protection, all four patients developed anti-72a1 antibody reactivity, with one patient experiencing a severe reaction upon the 7th infusion [37]. The authors of the study argued the need to humanize 72a1 for the therapy to be of future benefit.

The hu72a1 construct outlined herein displayed a 98% reduction in GAM reactivity as compared to m72a1, while maintaining comparable recognition of gp350 to that seen for ch72a1 and for m72a1 (Figure 3 and Figure 4). We also noted that hu72a1, like ch72a1 and m72a1, recognized the gp350 mimetic peptide P2, sequences DDRTLQL-A-QNPVYLIPETVPYIKWDN (Figure 5). This mimetic peptide was previously shown to bind to m72a1 and block m72a1 recognition of gp350 [33]. Humanization of m72a1 did not appear to alter virus neutralizing activity. Hu72a1 and ch72a1 inhibited P3HR1 superinfection of Raji cells by 79% and 74%, respectively (Table 1).

While overall characteristics and desirable properties of m72a1 were maintained in hu72a1, we did note differences in how hu72a1 interacts with gp350 when compared to m72a1. Hu72a1 antibody did not block m72a1 access of gp350 (Figure 6). An in silico examination of gp350 interaction with m72a1 VR and hu72a1 VR suggests that subtle changes may have been introduced to the 72a1 antibody binding site which could affect the antibody’s overall interaction with gp350. Models of gp350:72a1 interfacing amino acids predict that humanization of 72a1 might have strengthened or weakened individual amino acid contacts in gp350 common to mouse and human 72a1 antibody (Figure 7). These noted changes in gp350 contacts may explain why hu72a1 binds stronger to P2 as compared to m72a1 or ch72a1 (Figure 5), and why it was incapable of blocking m72a1 binding to gp350 (Figure 6). It would also appear that 72a1 antibody neutralization does not require a strict set of gp350 amino acid contacts.

In silico modeling predicts an overall preservation of CDR positioning, but hu72a1 HC CDR2 → LC CDR3 distance decreased by ~6 Å and hu72a1 HC CDR1 → HC CDR2 distance decreased by ~4 Å (Table 2). These changes in CDR distance may change H-bonds within the CDR from ones that were mostly covalent into ones that are more electrostatic. Spatial angles of LC CDR2 and HC CDR1 also changed by 10 and 7.4 degrees, respectively (Table 3 and Figure 1B). Lastly, the HC CDR3 loop that forms a bulge in m72a1 appeared slightly distorted in our hu72a1 model such that the tandem arginines in the CDR3 sequence AGGLRRVNWFAY are now shifted from CDR’s loop apex toward the rightward bulge position (Figure 1B). As the heavy chain CDR3 has the greatest effect on antibody binding energetics [46], it will be necessary to change the neighbouring framework amino acid sequences or re-sculpt 72a1 HC CDR3 to obtain a CDR3 shape which better reflects that of m72a1.

In summary, humanization of m72a1 did not change the ability of hu72a1 to recognize gp350 or the gp350 neutralization epitope contained in P2, or drastically alter virus neutralization. Whether subtle changes introduced during the humanization process and observed in our biochemical assays (Figure 5 and Figure 6) or noted in our in silico models masked a yet undiscovered mechanism of EBV neutralization by hu72a1 remains to be examined.

This work provides further structural and immunological characterization of hu72a1’s interaction with EBV gp350 and better defines 72a1’s virus neutralization properties. While testing in an appropriate animal model will be required to advance the hu72a1 antibody to clinical trials in transplant patients, the availability of comparative neutralization data between the original 72a1 hybridoma and the humanized counterpart is critical to better position the hu72a1 antibody for further development as an anti-EBV therapeutic.

## 4. Materials and Methods

### 4.1. Cell Culture and EBV Production

Anti-gp350 hybridoma m72a1 [24], virus producing cell lines P3HR-1 [50] and B95-8 [51], and Burkitt lymphoma line Raji [52] were maintained in RPMI-1640 (Wisent Bioproducts, St-Bruno, QC, Canada) supplemented with 10% heat-inactivated FBS and antibiotics. HEK 293 cells, used to express human and chimeric 72a1 antibody or soluble gp350/gp220 (CRL-1573, American Type Culture Collection (ATCC), Manassas, VA, USA) were maintained in DMEM supplemented with non-essential amino acids (Sigma, Aldrich; St Louis, MO, USA) and 10% FBS. The Chinese hamster ovary cell line CHO-K1 (CCL-61, ATCC) containing pcDNA3.1:gp350 ORF [53] and expressing gp350/220 on the cell surface was grown in Kiaghn’s F-12K medium supplemented with 10% FBS and antibiotics.

Virus stocks of enriched P3HR-1 virus were obtained from producer P3HR-1 cells cultured for 5 days in RPMI 1640 medium, 2% FBS and 30 ng/mL TPA (12-O-tetradecanoylphorbol-13-acetate, Sigma-Aldrich, St Louis, MO, USA). Spent culture medium was removed of cells and debris by centrifugation at 1500 rpm for 20 min, followed by centrifugation at 3000 rpm for 30 min. Culture medium was filtered through a 0.45 micron filter and the virus was concentrated by centrifugation at 30,000× *g* for 2 h at 4 °C. Virus pellets were aliquoted as 100× concentrates in serum-free RPMI and stored at −80 °C.

### 4.2. Construction of Chimeric and Humanized 72a1

The murine 72a1 HC partial coding sequence (GenBank KT211017.1) [34] was modified by addition of the *Bam*HI restriction enzyme recognition site upstream of the HC VR ATG start sequence and the introduction of a *Sca*I site at nucleotide position 409 (DNA sequence S1). The murine 72a1 lambda LC partial coding sequence (GenBank: KT211018.1) [34] was modified to remove an internal *Sca*I restriction enzyme site at nucleotide position 133 and restriction enzymes *Hind*III and *Hinc*II introduced upstream of the LC ATG start sequence and at nucleotide 372, respectively (DNA sequence S2). Nucleotide modification to introduce new restriction enzyme sites was not expected to change the 72a1 protein sequences. Modified m72a1 VR coding sequences were synthetically manufactured (gBlocks, Integrated DNA Technology, IDT, Coralville, IA, USA). The modified murine HC and LC sequences were ligated in-frame to human cDNA sequences encoding the human IgG1 HC and kappa LC constant regions and placed into pcDNA3.1 and pHook-3 expression vectors for expression of ch72a1 [33]. Construction of the hu72a1 antibody employed BLAST comparison scans (https://blast.ncbi.nlm.nih.gov, Nov. 21, 2016) of 72a1 HC and LC CDRs and their intervening framework protein sequences with human Ig homologs found in the National Center for Biotechnology Information, USA, protein databank. Human Ig VRs viewed as most similar to and most accommodating of m72a1 CDRs and framework sequences were designed and re-scanned to remove or “de-immunize” any residual murine-specific Ig protein sequences [47]. Final hu72a1 VR design was synthetically manufactured to allow for in-frame *Sca*I (DNA sequence S3) and *Hinc*II (DNA sequence S4) ligation into their respective human HC and LC constant regions and placement into pcDNA3.1 and pHook-3 expression plasmids [33]. G418/Zeocin resistant HEK 293 cell clones found to express high levels of anti-gp350 and anti-human Ig immunoreactivity were used to purify ch72a1 and hu72a1 IgG by protein A affinity chromatography [54]. m72a1 IgG was purified from spent hybridoma medium by protein G affinity chromatography [54]. IgG protein purity and concentration were measured by SDS-PAGE and by the Bradford protein assay using purified mIgG (Sigma-Aldrich) or purified hIgG as standards.

### 4.3. Construction and Purification of Soluble gp350 and gp220 Ectodomains

Gp220 ectodomain open reading frame (ORF) was obtained from CHO-K1 cells expressing pcDNA3.1:gp350gp220 RNA using first-strand cDNA synthesis kit (Sigma-Aldrich) and *pfx* PCR amplification with *Bam*H1-gp350-ATG sense primer 5′-gactcggatcc-ATGGAGGCAGCCTTGCTTGT GTGTCAGTACA-3′ (IDT DNA, Coralville, IA, USA) and *Sca*I-gp350 antisense 5′-CCACTGC AGTACTAGCATGGAGAG-3′. gp350 ectodomain was achieved after introduction of a silent *Sca*I restriction site at gp350 amino acid 463 and *Sca*I in-frame introduction of a synthetic gp350 no-splice (NS) optimized DNA sequence (gBlocks, IDT, Sequence S5). The gp350 NS construct encodes the EBV gp350 RNA splice donor/splice acceptor mutants [55]. Both gp220 and gp350 (NS) ORFs were ligated in-frame to a synthetic DNA fragment which encodes an optimized thrombin recognition site GGLTPRGVRLGG and a 6xHis-tag coding sequence flanked by *Sca*I and *Xba*I restriction sites (IDT DNA, Sequence S6) [56]. Final ectodomain constructs were ligated into pcDNA3.1 (Thermo Fisher Scientific, Waltham, MA, USA) allowing G-418 drug selection. HEK 293 cells were transfected with *Bgl*2 linearized expression plasmids and Lipofectamine 2000 (Thermo Fisher Scientific) followed by G-418 (Wisent) selection and colony screening for 72a1 antibody immunoreactivity. Soluble gp350 or gp220 ectodomains were purified from HEK 293 cells which were fed daily with a defined medium consisting of equal parts DMEM and Opti-MEM (Fisher-Thermo), and supplemented with SITE (Sigma-Aldrich) and antibiotics. After a 25-min centrifugation at 3000 rpm to remove cells and debris, the medium was buffer-exchanged by dialysis in Ni-affinity column wash buffer (20 mM phosphate buffer, 300 mM NaCl, 1 mM imidazole, pH 8.0). gp350- and gp220-containing preparations were cycled through His-Select Ni-affinity gel (Sigma-Aldrich) followed by column wash buffer and gp350 or gp220 elution in five column volumes of elution buffer (20 mM phosphate, 300 mM NaCl, 250 mM imidazole, pH 8.0). Ni-column eluate that was concentrated, and buffer-exchanged using an Amicon Ultra-4 centrifugal filtration unit (Sigma-Aldrich) was then loaded onto two tandem Sepharose 6 columns (Pharmacia, Stockholm, Sweden). Column fractions exhibiting peak m72a1 immunoreactivity were analyzed for purity by SDS-PAGE [57]. Fractions containing single bands of gp350 or gp220 were pooled and buffer-exchanged in Amicon Ultra-4 centrifugal filtration units and final protein concentration measured using the Bradford microprotein assay (Sigma-Aldrich) and BSA as a standard. gp350 preparations for ELISA were stored at 4 °C and used within 1 month.

### 4.4. gp350 ELISA

Purified gp350 or gp220 ectodomains, suspended at 0.1 μg/mL in PBS, were added as 100 μL aliquots to 96-well ELISA plates (Ultident, St. Laurent, QC, Canada). gp350 mimetic peptides comprising the major EBV neutralizing epitope [33] were synthesized and HPLC purified to >70% (Elim Biopharmaceuticals, Hayward, CA, USA). The peptides were resuspended at 2.5 μg/mL in PBS prior to overnight incubation in a 96-well ELISA plate at 4 °C as 100 μL aliquots. gp350/220 or peptide-coated plates were washed with PBS: 0.05% Tween-20 and blocked with 1% BSA:PBS prior to addition of antibody. After a 1-h incubation at 37 °C, bound antibody was detected using species affini-pure biotin-conjugated goat anti-mouse IgG or species affini-pure anti-human IgG in conjunction with horseradish peroxidase (HRP)-conjugated streptavidin (Jackson ImmunoResearch Laboratories, West Grove, PA, USA). Plates were color-developed using 3,3′ 5,5′-tetramethylbenzidine substrate (TMB solution, Bioshop, Burlington, ON, Canada). Colorization was stopped with an equal volume of 1N HCl, and ELISA wells read at 450_nm_/570_nm_. 72a1 competition assays were performed as previously described [33]. Plates were incubated for 2 h with serial dilutions of purified mIgG, m72a1, huIgG, ch72a1 or hu72a1 antibody prior to addition of biotin-conjugated m72a1 antibody [58].

### 4.5. gp350 Fluorescence and FACS Analysis

CHO-K1 cells or CHO-K1 cells expressing cell surface gp350 and gp220 were gently removed from tissue culture plates using versine, and washed several times with PBS prior to preparation of slides and fixing in methanol. Slides were incubated with m72a1, ch72a1 or hu72a1, or with their respective isotype-matched mouse or human antibody and developed with affini-pure biotin-conjugated goat anti-mouse IgG or anti-human IgG in conjunction with DTAF-streptavidin (Jackson ImmunoResearch Laboratories, West Grove, USA ). Anti-gp350 reactivity against B95-8 (Type I) and P3HR-1 (Type II) virus-carrying cells was determined by FACS analysis of TPA-treated B95-8 and P3HR-1 cell cultures. In preparation for FACS analysis, cells were incubated at 4 °C for 1 h with the 72a1 preparations, followed by three washes and incubation with the respective FITC-labeled secondary antibody. They were fixed in a 2% paraformaldehyde:PBS solution prior to FACS analysis.

### 4.6. EBV Antibody Neutralization Assay

Antibody neutralization potency was measured as the inhibition of EBV early antigen (EA) expression upon super-infection of Raji cells by P3HR-1 virus [43]. Stocks of concentrated virus (~5 × 10^4^ EA-inducing units) and purified antibody were initially incubated in a final volume of 0.1 mL for 60 min at 4 °C prior to the addition of 0.1 mL of 1 × 10^5^ Raji cells. Cells were incubated for 60 min at 4 °C followed by three washes with RPMI medium and cultured for 2 days in complete RPMI medium. Raji cells were then washed and spread on glass slides, followed by fixation in ice-cold methanol for 10 min. EA-expressing cells were revealed by indirect immunofluorescence microscopy using increasing dilutions of high EA-titer human serum and FITC-labeled goat anti-human IgG [59]. The % inhibition was calculated as [1-(Antibody EA+/Virus EA+)] × 100 following subtraction of EA levels in untreated cells.

### 4.7. In Silico Analysis

The 3D models of the mouse and humanized 72a1 antibody VRs were generated by the Rosetta Antibody Protocol http://rosie.graylab.jhu.edu/antibody [60,61,62,63,64]. 72a1 antibody:gp350 dockings were initially aligned using HEX 8.0 Cuda software [65] and further refined using the Rosetta SnugDock protocol http://rosie.graylab.jhu.edu/snug_dock [63,66,67] or the Rosetta Docking 2 Protocol http://rosie.graylab.jhu.edu/docking2 [63,66,68]. The level of humanization for the hu72a1 VR construct was assessed using the SHAB server database http://www.bioinf.org.uk/abs/shab [39]. Rosetta generated antibody, least-energy gp350:72a1 docking models, H-bond and protein:protein interface were evaluated using PyMOL Molecular Graphics System Version 2.01 Software, Schrödinger, LLC.

## 5. Conclusions

Transplant patients who experience acute EBV infection when their immune system is highly compromised are at risk of developing PTLD or other EBV-driven cancers. Prophylactic use of an anti-EBV neutralizing antibody during the period of high vulnerability is proposed to be an effective strategy in preventing EBV infection and related cancers. In vitro comparison testing of a humanized anti-gp350 antibody with its chimeric counterpart showed comparable virus neutralizing activity without the anti-mouse IgG immunoreactivity. These data support the use of humanized 72a1 as a platform launch point for future EBV therapeutic antibody designs.

## Figures and Tables

**Table 1:** Inhibition of in vitro EBV infection by chimeric and humanized 72a1.

Exp’t	UT ^1^	V ^1^	V + ch72a1 ^1^	V + hu72a1 ^1^	ch72a1 % Inhibition	hu72a1 % Inhibition
1	0	25.9	2.3	3.5	91.1	86.5
2	0.2	2.1	0.3	0.2	85.7	90.5
3	0.2	6.9	3.8	2.7	44.9	60.1
Average	0.1	11.6	2.1	2.1	73.9	79.0

^1^ % early antigen (EA) positive cells. UT, untreated; V, virus-infected; Antibody concentration of 1 μg/mL.

**Table 2:** CDR distances in human and mouse 72a1.

CDR Pairing	m72a1	hu72a1	∆H-Bonds ^1^
HC CDR1 → HC CDR2	13.9	11.6	2
HC CDR2 → LC CDR3	22.9	20.3	3
LC CDR3 → LC CDR1	11.7	12.4	1
LC CDR1 → LC CDR2	16.1	16.5	0
LC CDR2 → HC CDR3	7.4	8.3	1
HC CDR3 → HC CDR1	15.6	15	1

^1^ 1 unit = 1 H-bond distance (~2 Angstroms, Å) and calculated from mid-CDR amino acid α-carbon.

**Table 3:** CDR-loop-anchor distances and loop angular configuration for human and mouse 72a1.

72a1 VR	CDR	CDR AA	CDR AA Loop Termini C-α Carbon Distance *			CDR AA Loop Angle		
			CDR Anchor AA	Human	Mouse	∆ABC	Human	Mouse
LC VR	CDR1	RSSTGAVTTSNYAN	R N	12.2	12.5	RVN	79.8	77.3
	CDR2	GGTNNRVP	G P	10.4	10.8	GNP	98.1	87.9
	CDR3	VLWHSNHW	V W	6.3	6.3	VSW	27.5	24.7
HC VR	CDR1	KASGSSFTDYTMN	K N	12.9	11.9	KFN	61.7	69.1
	CDR2	LINPYNGGTR	G R	5.4	5.5	GYR	20.2	19.7
	CDR3	AGGLRRVNWFAY	A Y	5.8	5.5	ARY	20.6	23.3

LC VR, light chain variable region; HC VR, heavy chain variable region; CDR, complementarity determining region; AA, amino acid; * Distance measured in Angstroms (Å); CDR with even amino acids vertex = *n* + 1.

## Supplementary Materials

The following are available online at http://www.mdpi.com/2072-6694/10/4/112/s1, DNA sequence S1: *Bam*H I-chimeric HC VR-*Sca*I, DNA sequence S2: *Hind*III-chimeric 72a1 LC VR *Hinc*II, DNA sequence S3: *Bam*HI-human HC VR-*Sca*I, DNA sequence S4: *Hind*III-human LC VR-*Hinc*II, Sequence S5: gp350 no splice optimized sequence, sequence S6: EBV gp350 LVL-thrombin recognition site GG-LTPRGVRL-GG and a 6xHis-tag HHHHHH-G coding sequence flanked by *Sca*I and *Xba*I restriction sites, Figure S1: gp350 immunofluorescence for chimeric (ch)72a1 constructed with GenBank heavy and light chain variable regions GenBank KT211017.1 and KT211018.1 or GenBank KP863467.1 and KP863466.1.
