# Supplementary material for: Construction and Characterization of a Humanized Anti-Epstein-Barr Virus gp350 Antibody with Neutralizing Activity in Cell Culture
Source: Cancers (Basel). 2018 Apr 9;10(4):112. doi: 10.3390/cancers10040112 (PMC5923367; doi:10.3390/cancers10040112)
Supplement: Supplementary file 1 [file cancers-10-00112-s001.pdf]

Supplementary Materials: Construction and Characterization of a Humanized Anti-Epstein-Barr Virus gp350 Antibody with Neutralizing Activity in Cell Culture

Jerome E. Tanner, Jing Hu and Caroline Alfieri

DNA sequence S1. *Bam*HI-chimeric HC VR-*Sca*I

CCAGGGATCCATGGGATGGAGGTGGATCTTTCTCTTCCTCCTGTCAGGAACTGCAGGTGTCC
ACTCTGAGGTCCAGCTGCAACAGTCTGGACCTGAACTGGTGAAGCCTGGAACTTCAATGAA
GATATCCTGCAAGGCTTCTGGTTCCTCATTCACTGACTACACCATGAACTGGATGAAGCAGA
GCCATGGAAAGAACCTTGAGTGGATTGGACTTATTAATCCTTACAATGGTGGTACTAGGTAC
AACCAGAAGTTCAAGGGCAAGGCCACATTAACTTTAGACAAGTCATCCAGCACAGCCTAC
ATGGAGGTCCTCAGTCTGACATCTGAGGACTCTGCAGTCTACTACTGTGCAGGGGGATTGCG
ACGGGTAAACTGGTTTGCTTACTGGGGCCAAGGGACTCTGGTCTCTGTCAGTACTTCTGCA

DNA sequence S2. *Hind*III- chimeric 72a1 LC VR *Hinc*II

AAGCTTCCATGGCCTGGATTTCACTTATACTCTCTCTCCTGGCTCTCAGCTCAGGGGCCATTT
CCCAGGCTGTTTTGACTCAGGAATCTGCACTCACCACATCACCTGGTGAAACAGTCACACTC
ACTTGTCGCTCAAGCACTGGGGCTGTCACAACTAGCAACTATGCCAACTGGGTCCAAGAAA
AACCAGATCATTTATTCACTGGTCTAATAGGTGGTACCAACAACCGAGTTCCAGGTGTTCCT
GCCAGATTCTCAGGCTCCCTGATTGGAGACAAGGCTGCCCTCACCATCACAGGGGCACAGA
CTGAGGATGAGGCAATATATTTCTGTGTTCTATGGCACAGCAATCATTGGGTGTTCGGTGGA
GGAACCAAGTTAACACTGACTGTCCTA

DNA sequence S3. *Bam*HI-human HC VR-*Sca*I

CCAGGGATCCATGGGGTGGCGCTGGATATTCCTTTTCTTGCTTAGCGGCACTGCCGGGGTAC
ATTCCCAGGTCCAGCTGGTCCAATCTGGTGCCGAAGTTAAAAAACCTGGTGCATCTGTGAA
GGTCAGCTGCAAGGCATCTGGTTCATCCTTCACAGACTACACTATGAACTGGGTTCGCCAAG
CGCCCGGCCAGGGTTTGGAATGGATGGGCCTGATAAACCCGTACAACGGTGGCACTCGGTA
TAACCAGAAATTTAAGGGCAGAGTCACAATGACCCGCGACACCTCCACCAGCACCGTGTAT
ATGGAACTGTCCTCTCTGCGGTCCGAGGACACAGCGGTGTACTATTGCGCTGGAGGGCTCC
GGCGCGTCAACTGGTTTGCCTATTGGGGCCAAGGCACACTTGTTAGCGTGAGTACTTCTGCA

DNA sequence S4. *Hind*III-human LC VR-*Hinc*II

AAGCTTCCATGGCGTGGATTTCATTGATCCTTTCTCTGCTCGCGCTGTCATCTGGTGCTATAT
CTCAGACGGTTGTCACCCAAGAGCCTTCCCTGACAGTGTCTCCGGGGGGTACGGTAACGCT
GACTTGCAGATCATCTACGGGCGCAGTAACTACATCCAACTATGCGAATTGGTTTCAACAG
AAGCCCGGTCAAGCCCCGCGCACCTTGATAGGGGGCACAAACAACCGAGTTCCGGGGGTC
CCGGATCGGTTTTCTGGCAGTATACTTGGGAATAAGGCCGCACTGACAATTACAGGGGCAC
AGGCCGATGATGAGTCCGACTACTATTGCGTCCTGTGGCACTCTAATCATTGGGTGTTTGGT
GGTGGAACCAAGTTAACACTCACGGTTCTT

Sequence S5. Gp350 no splice optimized sequence

CCTGCAAGTACTGGCCCCACTGTAAGTACCGCCGACGTAACGTCTCCCACACCAGCGGGGA
CAACATCCGGCGCCTCTCCCGTGACTCCATCCCCATCCCCCTGGGACAACGGAACAGAGTC
TAAGGCACCAGATATGACCAGCAGCACATCTCCCGTAACGACGCCTACGCCCAATGCCACG
TCTCCTACACCGGCTGTAACTACCCCCACGCCGAACGCAACCTCCCCGACTCCCGCGGTGA
CGACCCCTACCCCGAATGCTACAAGCCCAACATTGGGAAAGACCTCACCTACTTCTGCTGTT

ACGACGCCAACGCCGAACGCAACTTCACCCACCCTGGGTAAGACAAGTCCCACGTCAGCT
GTCACCACTCCAACCCCCAATGCCACTTCCCCGACTCTCGGGAAAACAAGTCCTACCTCTGC
AGTTACTACACCCACTCCAAACGCAACGGGACCAACGGTGGGCGAAACCAGTCCACAAGC
GAACGCCACCAACCACACGCTGGGCGGTACCTCACCGACACCAGTAGTCACATCCCAACCT
AAGAACGCGACCTCCGCGGTCACTACCGGCCAACATAACATTACATCCTCCAGCACTTCAT
CTATGTCATTGCGGCCGTCTTCCAACCCTGAAACTCTTTCCCCTAGTACAAGCGATAATTCTA
CTAGCCATATGCCGCTCTTGACATCCGCCCACCCAACGGGCGGGGAAAACATTACACAGGT
GACCCCAGCTTCCATAAGCACACACCATGTGAGCACCAGCAGTCCAGAACCTCGCCCTGGG
ACGACGTCCCAGGCGAGTGGACCAGGCAACTCATCTACCTCTACAAAACCTGGTGAAGTCA
ACGTGACAAAAGGCACTCCGCCCCAGAATGCAACGAGTCCACAGGCCCCTAGCGGACAGA
AAACTGCCGTTCCAACTGTAACTTCTACCGGGGGAAAAGCGAACTCAACTACAGGAGGAA
AGCACACTACAGGGCATGGGGCAAGGACCAGCACAGAACCGACTACTGATTATGGAGGGG
ATAGCACGACGCCCAGGCCCAGATACAACGCAACCACTTATCTTCCACCCTCAACGTCAAG
TAAGCTCAGGCCGAGGTGGACGTTCACGAGTCCACCCGTAACTACCGCCCAAGCTACAGTT
CCAGTCCCGCCTACCTCACAACCCCGATTCAGCAATTTGTCTAACCTCTCCATGGCTAGTAC
TGGAGGT

Sequence S6. EBV gp350 LVL-thrombin recognition site GG-LTPRGVRL-GG and a 6xHis-tag HHHHHH -G coding sequence flanked by *Sca*I and *Xba*I restriction sites

agtactGGGAGGCTTAACACCAAGAGGTGTAAGACTAGGTGGACATCACCATCACCACCATGG
GTGATAGGGtctagacc

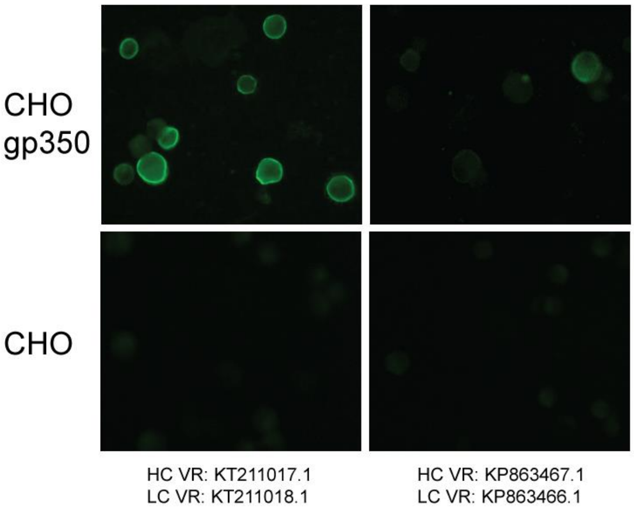

Figure S1. Chimeric 72a1 reactivity to gp350 expressed on the surface of CHO cell clone 5.20 (CHO gp350) or parental CHO-K1 cell line (CHO). Chimeric 72a1 antibody was constructed from GenBank heavy and light chain variable regions KT211017.1 and KT211018.1 (left column) or GenBank KP863467.1 and KP863466.1 (right column). Ch72a1 antibody was detected with biotinylated affinipure goat anti-human serum and DTAF-streptavidin.

© 2018 by the authors; licensee MDPI, Basel, Switzerland. This article is an open access article distributed under the terms and conditions of the Creative Commons by Attribution (CC-BY) license (http://creativecommons.org/licenses/by/4.0/)
